# Concerns about Methodological Flaws and Use of Retracted Evidence in the Opinion on COVID-19 Vaccination and Excess Mortality in Japan

**DOI:** 10.31662/jmaj.2025-0440

**Published:** 2025-11-14

**Authors:** Satoshi Kutsuna, Sadao Suzuki

**Affiliations:** 1 Department of Infection Control and Prevention, Graduate School of Medicine, Center for Infectious Disease Education and Research, Osaka University, Suita, Osaka, Japan; 2 Department of Public Health, Nagoya City University Graduate School of Medical Sciences, Nagoya, Japan

**Keywords:** excess mortality, COVID-19 vaccination, retracted study, epidemiology, risk communication

## To the Editor,

We read with great concern the recent opinion article published in the *JMA Journal* ^(1)^, which attributes excess mortality in Japan in 2023 largely to coronavirus disease 2019 (COVID-19) vaccination. While we respect academic freedom and open scientific debate, we are deeply troubled by the methodological and interpretive flaws in this article. Left uncorrected, its claims risk misleading readers and undermining the credibility of this journal, as well as the public’s trust in evidence-based medicine.

The first problem concerns study design. The article presents descriptive mortality statistics as though they were capable of establishing a causal association. This impression is reinforced by the article’s very title, which misleadingly suggests a direct causal link between COVID-19 vaccination and excess mortality in 2023. Descriptive epidemiology can only generate hypotheses, not test them, and causal inference requires individual-level data and appropriate analytical methods. Even at the ecological level, no correlation coefficients or association measures were provided; instead, mortality trends and vaccination numbers were merely juxtaposed, creating the impression of a causal link. This is a textbook example of ecological fallacy, a limitation long recognized in epidemiology ^(2)^. Without individual-level data linking vaccination status to outcomes, causal inference cannot be made.

Second, the analysis itself is inconsistent. The authors repeatedly equate “excess mortality” with “increased mortality rate,” though these are conceptually distinct. Excess mortality refers to the difference between observed and expected deaths, and its estimation is highly sensitive to the definition of baseline and the method used. Without sensitivity analyses to account for these assumptions, such estimates remain unstable and prone to misinterpretation.

Furthermore, the article misuses data from the Health Center Real-time Information-sharing System on COVID-19 (HER-SYS). In this system, cases in which vaccination dates were not recorded were automatically counted as “unvaccinated.” The Ministry of Health, Labour and Welfare explicitly warned that such misclassification must not be used for evaluating vaccine effectiveness ^(3), (4)^. This erroneous treatment of missing data created the misleading appearance that vaccinated individuals had a higher risk of infection. Building such a conclusion on biased data is methodologically flawed and scientifically invalid.

Third, the discussion invokes speculative biological mechanisms to explain supposed increases in cancer or cardiovascular mortality, citing phenomena such as immunoglobulin G4 class switching or nuclear entry of spike protein. These references are drawn from a limited set of experimental studies and do not provide any epidemiological confirmation at the population level. Laboratory observations of molecular pathways cannot be extrapolated to explain increases in nationwide mortality statistics, and the article fails to present any supporting data from large-scale epidemiological studies. Relying solely on selective citation of mechanistic reports while ignoring robust population-based evidence is scientifically unsound. Cancer in particular has a long natural history, typically requiring many years from the initial cellular changes to clinically apparent disease and eventual death. Most solid tumors progress over a period of several years to decades before leading to mortality. The suggestion that third-dose vaccination in 2022 could cause a measurable increase in cancer deaths within the same year is therefore biologically implausible and incompatible with established oncological understanding ^(5)^. Moreover, the article cites as supporting evidence a source ^(6)^ that reflects the same arguments as the paper by Gibo et al. ^(7)^, which was later published in Cureus and has since been formally retracted due to serious methodological flaws. The fact that the very study underpinning their claim of increased cancer mortality has been withdrawn underscores the scientific inappropriateness of relying on such evidence. Continuing to reference a retracted work without disclosure further undermines the credibility of the article’s argument. Moreover, other well-documented contributors to 2023 mortality were completely disregarded. For example, record-breaking heat waves during the summer led to a marked increase in heat-related deaths ^(8)^, and influenza resurged in an unusually early and intense season after years of suppression during the COVID-19 pandemic ^(9)^. In addition, Japan’s rapidly aging population contributes to a steady natural increase in annual deaths independent of any external shock ^(10)^. Ignoring these substantial and well-recognized drivers of mortality, while attributing excess deaths almost exclusively to vaccination, results in a distorted and misleading interpretation.

Recent analyses ^(11)^ that adjust for temperature and influenza have shown that a portion of excess mortality in Japan during 2022-2023 remains unexplained. However, it is epidemiologically incorrect to attribute this residual mortality solely to vaccination. Unexplained variance in excess deaths can reflect a wide range of factors, including delayed access to medical care, other infectious diseases, socioeconomic stressors, and reporting biases. To claim a causal role for vaccination, stronger evidence would be required, such as consistent age-specific patterns, dose-response relationships, or reproducibility across international settings. None of these criteria has been met. Therefore, attributing the unexplained fraction of excess deaths to COVID-19 vaccines is premature and scientifically unfounded.

Beyond these scientific flaws, it is also problematic that the article disregards the large body of high-quality epidemiological evidence consistently showing that COVID-19 vaccination reduces severe outcomes and mortality, including during the Omicron period ^(12)^. The cumulative effect is not simply an academic misstep. Publishing such interpretations in a respected medical journal has important implications for risk communication. Once in print, claims of this kind are rapidly disseminated on social media and are often cited by the public as “evidence” of vaccine harm. International experience shows that even when such papers are later corrected or retracted, their influence can persist in fueling vaccine hesitancy ^(13), (14)^. To prevent misunderstanding and protect public trust, it is desirable for the editorial board to provide clear scientific context or expert commentary alongside controversial pieces. By adopting such proactive measures, the journal can maintain its role as a trusted source of evidence-based medicine while ensuring that discussion remains balanced and scientifically sound.

In light of these problems, we urge *JMA Journal* to take proactive steps. Strengthening peer review of opinion pieces, especially those addressing socially sensitive topics such as vaccination, is essential. Reviewers should ensure that claims are supported primarily by peer-reviewed epidemiological studies and official statistics, rather than secondary sources or general-audience texts. Furthermore, when controversial views are published, editorial notes or expert commentaries should accompany them so that readers can immediately recognize the limitations of the arguments. By doing so, the journal would not suppress discussion but rather safeguard it, ensuring that debate remains anchored in scientific validity.

Scientific journals play a vital role in shaping professional and public discourse, and with this role comes the responsibility to safeguard scientific integrity and public trust. The article in question relies on unsupported hypotheses, misinterprets official data, and neglects well-established confounders. To ensure that *JMA Journal* remains a trusted forum for evidence-based medicine, we strongly urge the editorial board to acknowledge these flaws and take proactive steps to prevent similar occurrences in the future.

## Article Information

### Author Contributions

Drafting of the manuscript, literature review, and critical revision: Satoshi Kutsuna

Conceptualization, supervision, and critical revision for important intellectual content: Sadao Suzuki

### Conflicts of Interest

Satoshi Kutsuna has received lecture fees from Pfizer Inc. and has served as a scientific advisor for Pfizer Inc. on educational pamphlets. Sadao Suzuki declares no conflicts of interest.

### Sources of financial support

None

### Approval code issued by the institutional review board (IRB) and the name of the institution(s) that granted the approval

Not applicable

## References

[1] Kakeya H, Nitta T, Kamijima Y, et al. Significant increase in excess deaths after repeated COVID-19 vaccination in Japan. JMA J. 2025;8(2):584-6. 40416011 10.31662/jmaj.2024-0298 PMC12095670

[2] Greenland S. Ecologic versus individual-level sources of bias in ecologic estimates of contextual health effects. Int J Epidemiol. 2001;30(6):1343-50. 11821344 10.1093/ije/30.6.1343

[3] The Asahi Shimbun. Health Minist Misclassified Vaccin Data COVID. Published 2022. Available at: https://www.asahi.com/ajw/articles/14634078

[4] Horie Y, Ishiguro C, Mimura W, et al. Validity of vaccination information in the COVID-19 surveillance system in Japan: implications for developing efficient and highly valid data collection systems in future pandemics. GHM Open. 2024;4(2):84-90. 40144964 10.35772/ghmo.2024.01007 PMC11933969

[5] Hanahan D. Hallmarks of cancer: new dimensions. Cancer Discov. 2022;12(1):31-46. 35022204 10.1158/2159-8290.CD-21-1059

[6] Kojima S. COVID vaccine review Part 2, Kadensha; 2024. Japanese.

[7] Gibo M, Kojima S, Fujisawa A, et al. Retraction: increased age-adjusted cancer mortality after the third mRNA-lipid nanoparticle vaccine dose during the COVID-19 pandemic in Japan. Cureus. 2024;16(6):r143. 38933341 10.7759/cureus.r143 PMC11204115

[8] State of the climate in Asia 2023 [Internet]. World Meteorological Organization (WMO) Publication. 2024 [cited 2025 Sept 2]. Available from: https://wmo.int/publication-series/state-of-climate-asia-2023

[9] Wagatsuma K, Otoguro T. Changing seasonality of influenza in the post-COVID era in Japan. JMA J. 2024;7(1):138-9. 38314406 10.31662/jmaj.2023-0150 PMC10834215

[10] Current population estimates as of October 1, 2023 [Internet]. Statistics Bureau of Japan, Ministry of Internal Affairs and Communications. 2024 [cited 2025/10/23]. Available from: https://www.stat.go.jp/english/data/jinsui/2023np/index.html

[11] Devanathan G, Chua PLC, Nomura S, et al. Excess mortality during and after the COVID-19 emergency in Japan: a two-stage interrupted time-series design. BMJ Public Health. 2025;3(1):e002357. 40196438 10.1136/bmjph-2024-002357 PMC11973774

[12] Andrews N, Stowe J, Kirsebom F, et al. Covid-19 vaccine effectiveness against the omicron (B.1.1.529) variant. N Engl J Med. 2022;386(16):1532-46. 35249272 10.1056/NEJMoa2119451 PMC8908811

[13] Taros T, Zoppo C, Yee N, et al. Retracted Covid-19 articles: significantly more cited than other articles within their journal of origin. Scientometrics. 2023;128(5):2935-43. 37101974 10.1007/s11192-023-04707-4 PMC10089824

[14] Frampton G, Woods L, Scott DA. Inconsistent and incomplete retraction of published research: a cross-sectional study on Covid-19 retractions and recommendations to mitigate risks for research, policy and practice. PLoS One. 2021;16(10):e0258935. 34705841 10.1371/journal.pone.0258935 PMC8550405

